# Supplementary material for: Cortical depth profiles in primary visual cortex for illusory and imaginary experiences
Source: Nat Commun. 2024 Feb 2;15:1002. doi: 10.1038/s41467-024-45065-w (PMC10837448; doi:10.1038/s41467-024-45065-w)
Supplement: Supplementary file 1 — Supplementary Information [file 41467_2024_45065_MOESM1_ESM.pdf]

Supplementary Information

For the manuscript *"Cortical depth profiles in primary visual cortex for illusory and imaginary experiences"* by Johanna Bergmann, Lucy S. Petro, Clement Abbatecola, Min S. Li, A. Tyler Morgan and Lars Muckli

Supplementary Figures

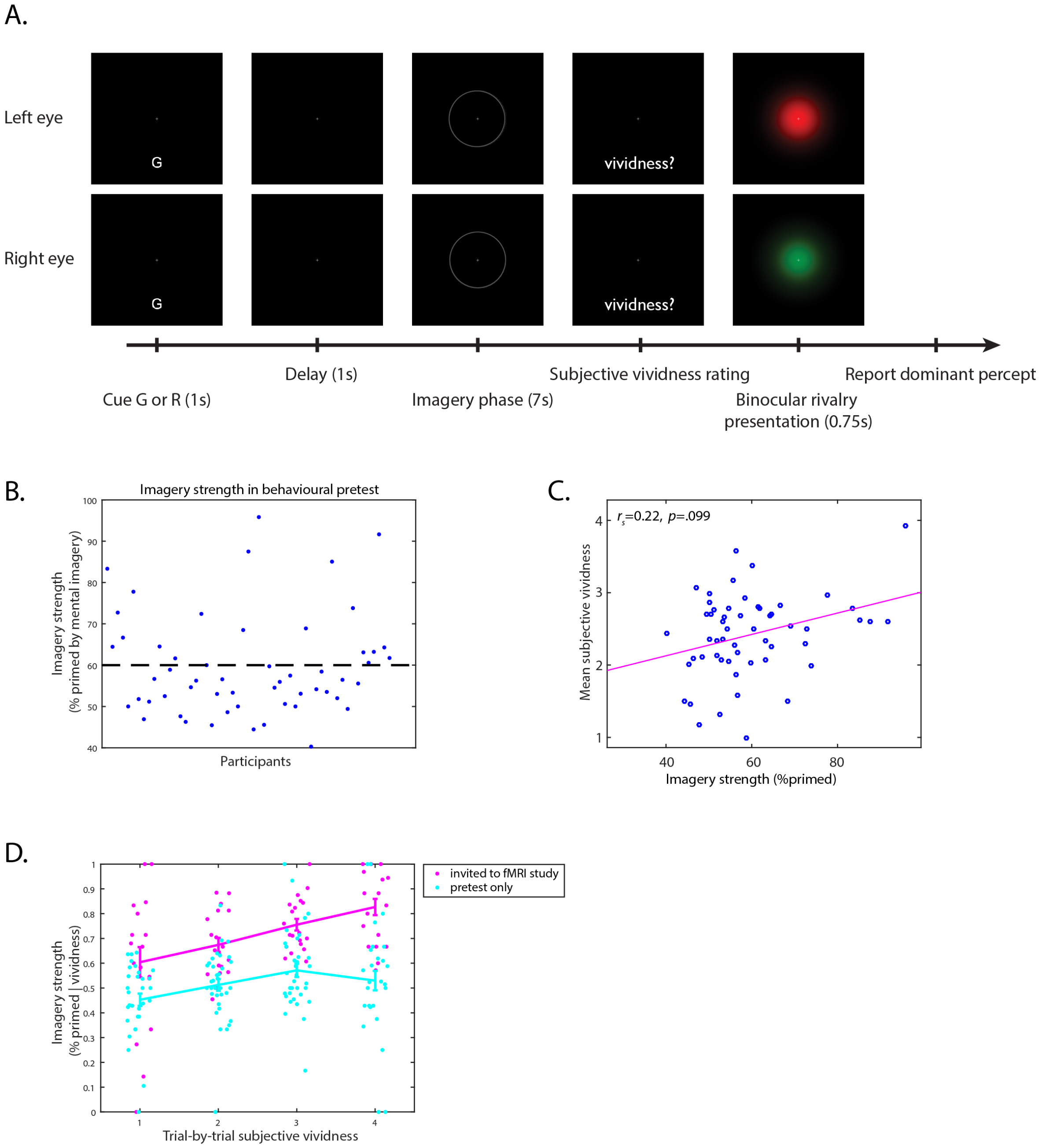

Supplementary Figure 1. Behavioural pretest procedure and analysis. A. The experimental procedure involved cueing participants to imagine a specified colour at the start of each trial. After a brief delay, participants had 7 seconds to imagine the colour, guided by a faint central circle indicating the visual field location. They then rated the subjective vividness on a scale from 1 to 4. Following this, a binocular rivalry stimulus, featuring Gaussian-windowed images of the two colours, was presented, one to each eye. Participants identified the dominant stimulus via button press. **B.** Only participants with an imagery strength (%primed) of 60% or higher were invited to the fMRI experiment. Data points denote individual participants; the dashed line indicates the 60% threshold. n=55 and n=52

participants took part in the behavioural pretest for experiment 1 and 2, respectively. Participants involved in both fMRI experiments (n=4) participated once in the behavioural pretest, and are only included in Experiment 1's pre-test figures. **C.** In line with previous findings[34], individual mean vividness and imagery strength did not correlate significantly with each other. Each data point denotes one participant; Spearman rank correlation was used. **D.** A relationship between vividness and imagery-induced priming can be observed on a trial-by-trial level. We split up the sample into those participants who were invited for the fMRI part of the study (pink dots), and those who were not (cyan dots). Error bars represent ±SEM. Replicating previous findings[34], a higher vividness rating in a given trial was associated with a higher probability of subsequent priming during binocular rivalry in Experiment 1 (significant main effect of vividness in a linear mixed effects model, beta = 0.17, 95%CI [0.04, 0.30], $t(142)=2.61$, $p = 0.010$). In Experiment 2, this effect was only present in the fMRI participants group, resulting in a significant interaction of group and vividness (beta = 0.38, 95%CI [0.10, 0.67], $t(145) = 2.64$, $p = 0.009$) but a non-significant main effect of vividness (beta = -5.32e-04, 95%CI [-0.13, 0.13], $t(145) = -7.90e-03$, $p = 0.994$). Not everyone used the whole vividness rating scale, resulting in different amounts of data per vividness rating.

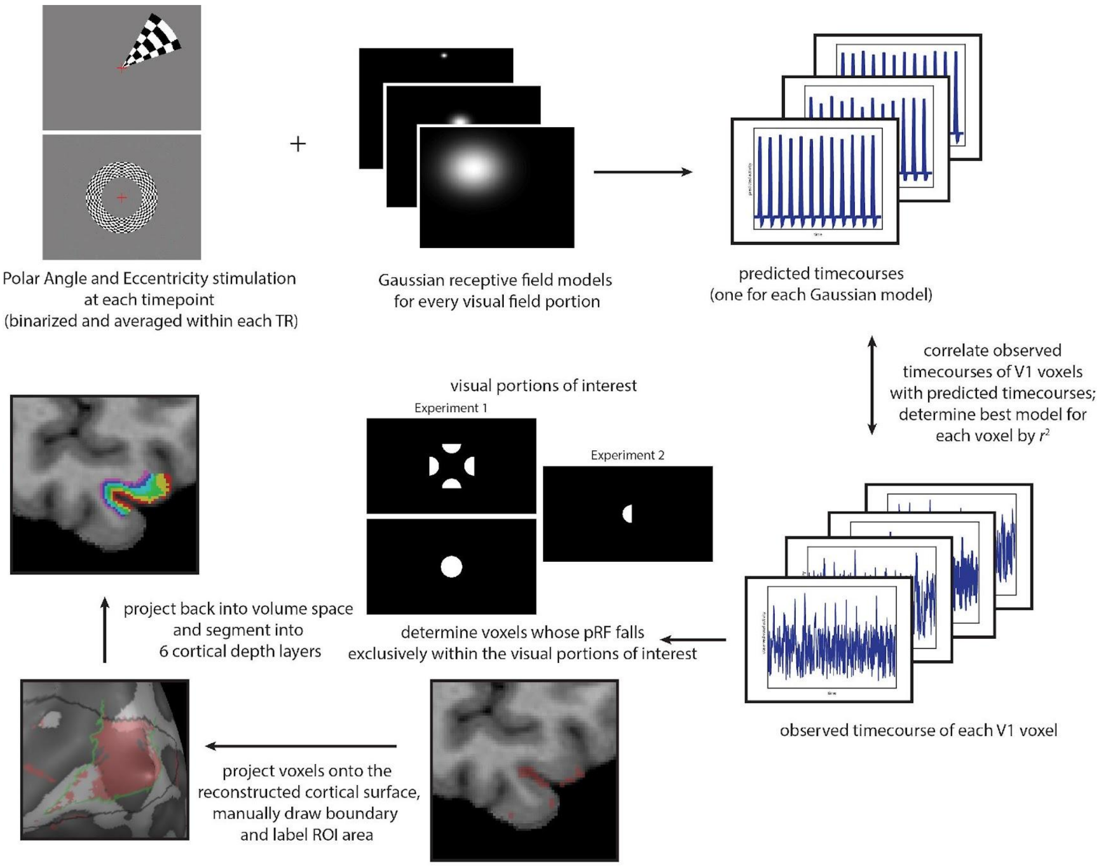

Supplementary Figure 2. Region-of-interest definition procedure. All participants underwent a retinotopic mapping procedure after the experimental task. This was used to 1) map the boundaries of V1, and 2) estimate population-receptive field (pRF) models. To obtain pRF models, information about the stimulus sequence during Polar Angle and Eccentricity stimulation is needed. For this purpose, we first generated binarized stimulus frames of the Polar Angle and Eccentricity stimulation for each timepoint, which were then averaged within each repetition time (TR). Next, we created Gaussian models of different sizes for every visual field portion. For each Gaussian model, we calculated how a voxel's response to the retinotopic mapping stimulation should look like over time, if it was responsive to the visual portion modelled by the Gaussian. These models were then correlated with the actual timecourses of V1 voxels. The Gaussian model that explained most of the voxel activity's variance (r^2) was then selected as the best model. With this approach, we could determine which voxels represented those portions of the visual field that we were interested in. To exclude excessive levels of noise, we set a threshold for r^2. The consequence of this is that most of the voxels above the threshold are close to the pial surface, where the overall BOLD activity strength is higher[27]. Further cross-checks confirmed the validity of the approach (see **Supplementary Fig. 3** and Methods). The voxels were then projected onto the cortical surface, and after cross-checking the voxels' location with the anatomy and the retinotopic maps to ensure the models were anatomically accurate, we manually drew boundaries around them and labelled the patches. Following this, the labelled patches of interest from the two hemispheres were projected back into volume space, where they were combined into one region of interest and segmented into 6 cortical depth layers.

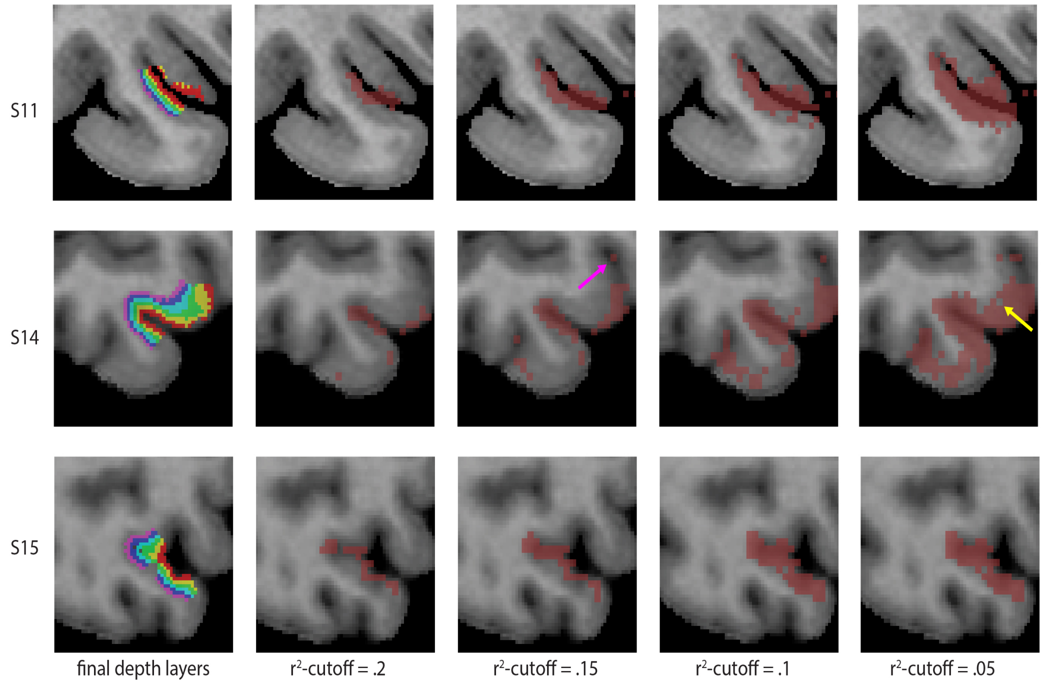

Supplementary Figure 3. Three experiment 1 subjects illustrating the influence of different r^2-thresholds on voxel selection (transverse view, right hemisphere). Subjects are represented in rows. Final cortical depth layers are in the left column, and preliminary region of interest (ROI) estimates (dark red voxels) with varying r^2-thresholds are in four columns to the right (thresholds decrease left to right). Population-receptive field models can be heavily affected by fMRI signal noise. As a result, some voxels' best model may fall within the visual areas of interest, even when it is evident from the anatomy that the voxels represent other portions of the visual field (e.g., see voxel indicated by the pink arrow in S14). To address this, a conservative approach using r^2-thresholding was employed, resulting in most remaining voxels being located near the pial surface, where the fMRI signal is strongest. To ensure all depth layers were represented nonetheless, we projected pRF-estimated voxels onto the cortical surface, created patches-of-interest, and segmented them into 6 cortical depth layers in volume space. This procedure is justified anatomically as neurons are organized in hypercolumns, which means that neurons located in the deeper layers process input from the same visual field portions as those stacked on top of them[64]. To validate this approach, we checked preliminary ROIs with lower r^2-cutoffs. More voxels located in deeper grey matter portions were added to the preliminary ROIs when the r^2-cutoff was lowered, confirming the validity of our approach. Voxels with the best model falling outside the visual field of interest due to noise were included in cortical depth layers based on anatomical considerations and cross-checks with retinotopic maps (e.g., see voxel indicated by the yellow arrow in S14). As another safety check, we also found that the pattern of SVM classification results remained consistent when excluding these voxels (data not shown). Note that in some participants, e.g. S11, the ROI spans both sides of the sulcus because it extends further transversally, where the portions from the two sides connect. Hence, it is not an accidental, distortion-induced 'spilling over' from one side of the sulcus to the other.

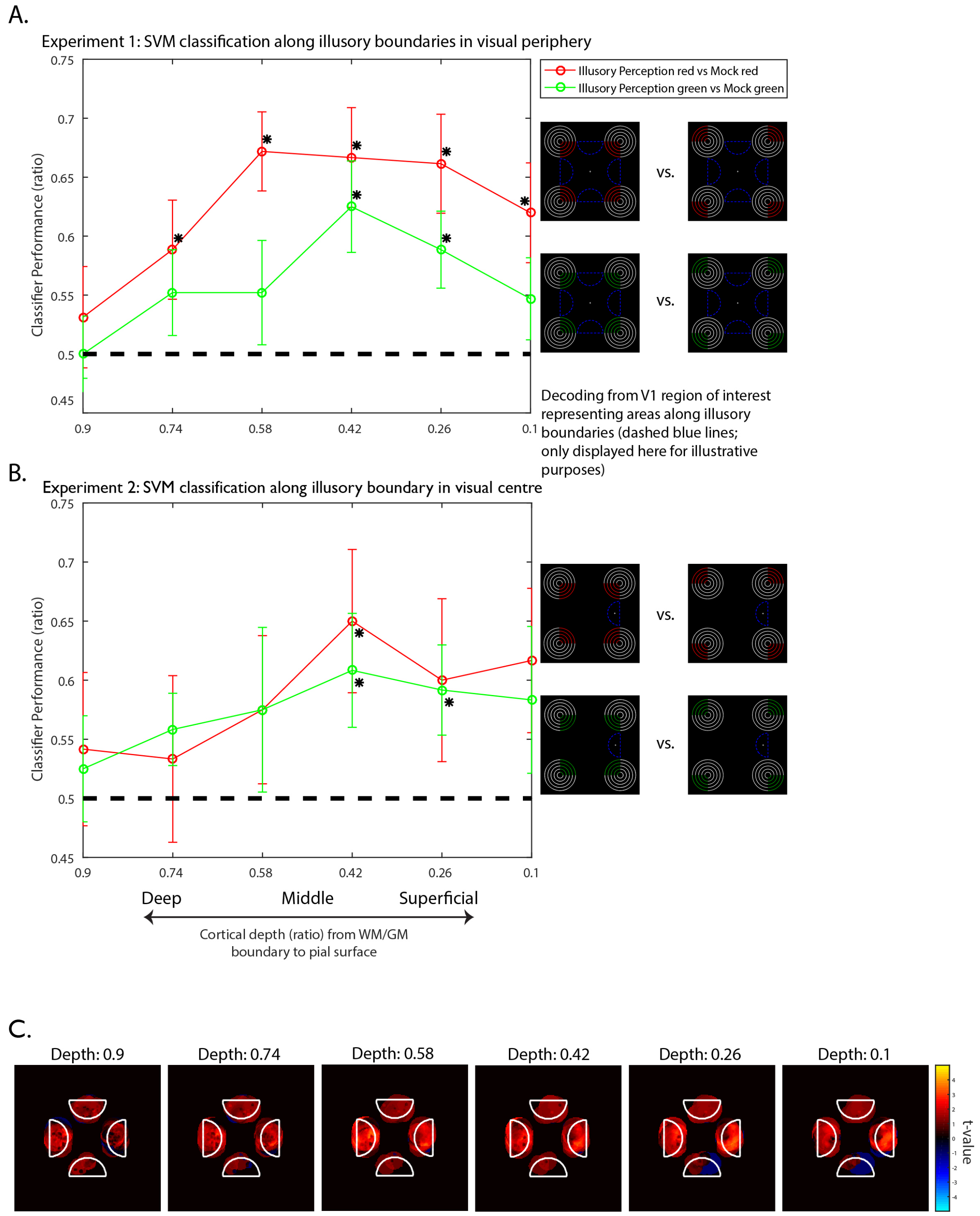

Supplementary Figure 4. Decoding of illusory perception vs. no illusory perception. A and B. To better compare our results to Kok et al.'s[7] univariate results, we decoded illusory perception against its mock version in experiment 1 (**A**, peripheral region of interest; n=16), and experiment 2 (**B**; n=10). For both experiments, we could run the analysis twice – for green and red stimuli. The dashed black line designates chance level; asterisks denote significant above-chance decoding (p_{adj} < .05, FDR-corrected). In experiment 1, decoding performance increased towards superficial depths, with significant decoding in both colours at cortical depths 2/3 (all p_{adj} < .009, see Methods section). This pattern largely replicated in experiment 2 (p_{adj} = .02, except for red at the 2nd depth, which was not significant), supporting our main finding that illusory color is decodable at the 2nd superficial depth. It

also matches findings that figure-ground edges and illusory contours selectively activate layer 2/3 in mice[21,23] and non-human primates[22], but contradicts Kok's et al.'s findings that illusory perception involves deep layers. **C.** Visual field projections. This approach translates voxel influence into visual space, highlighting which visual field portions (or pixels) contribute most to SVM classification. Shown here are the projections from decoding green illusory perception vs. its mock condition from experiment 1's peripheral ROI voxels (peripheral visual areas of interest outlined in white). More superficial depths are shown towards the right. Colour-encoded t-values indicate each pixel's influence on decoding across subjects. The head coil limited several participants' visibility in the lower/upper part of the screen, presumably leading to less voxel coverage and overall weaker t-values here. Note that statistical analyses were conducted with the decoding analyses (**A**), not on visual projections. If horizontal connections or activity spilling over from the ring-shaped inducers are responsible for our findings, we should expect that the most informative voxels represent portions closer to the inducers, which does not seem to be the case. Further, horizontal connections are present in all layers[65–67], and their visuospatial extent is larger in layer 4 and deeper layers[66,67]. This would predict a different laminar decoding pattern than what we observe.

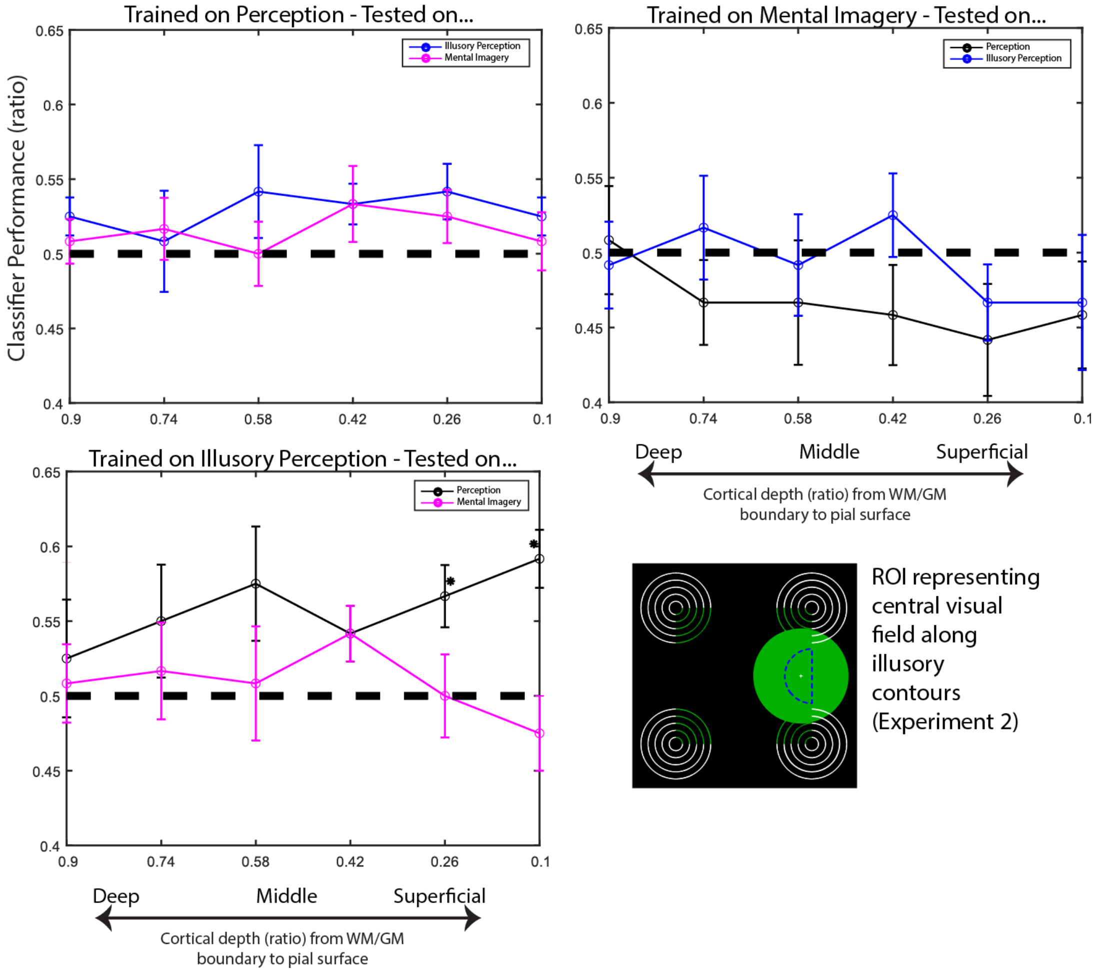

Supplementary Figure 5. Cross-classification of the three main conditions. An SVM classifier was trained to decode red vs. green in one condition (illusory perception, mental imagery, or perception), and then tested on the two other conditions. This was done for each cortical depth with the data of experiment 2, where the illusory contour, the perceptual and imagined stimuli were represented within the same V1 region of interest. The black dashed lines in the plots represent chance level; asterisks denote significant above-chance decoding (p_{adj}<.05, one-sided bootstrapping of the mean, multiple-comparison corrected); error bars represent ±SEM. Cross-classification, though more noise-sensitive than cross-validation, revealed significant decoding when training on illusory colour and testing on perceptual colour in the two most superficial layers (at depth 0.26, i.e. 2nd depth: $\hat{\mu}$ = 0.57, p_{adj} =.007, 90%CI [0.53, 0.6]; at depth 0.1, that is 1st depth: $\hat{\mu}$ = 0.59, p_{adj} < .001, 90%CI[0.56, 0.63]; see Methods section). The reverse – training on perceptual colour and testing on illusory colour - was not significant. This pattern that training on perception shows lower decodability has been observed previously[68]. It may be due to the fact that overall signal strength during perception is higher: As a result, the SVM classifier may be less sensitive to capture the colour-specific portions of information that it has in common with the illusory stimulus. For mental imagery, cross-classification was not significant, neither for illusory perception, nor for perception (p_{adj}<.05). Such significant cross-classification effects have been reported in previous studies, which used larger voxels and did not distinguish between different cortical depths[35,69]. When using smaller voxels and decoding by layer, voxels are less prone to pooling signals over a larger space and from a larger range of

cortical depths, so it may be that the (layer-wise) divergences between these two experiences become more apparent. However, with layer resolution data, Iamshchinina et al.[25] found cross-classification effects in a mental rotation task (see main manuscript for discussion).

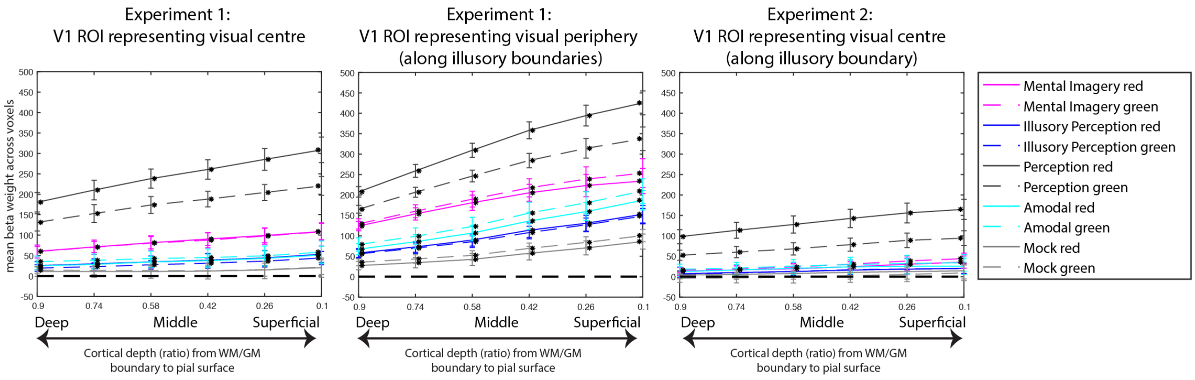

Supplementary Figure 6. fMRI activation levels by cortical depth layers of the V1 regions of interest of both experiments. Error bars denote ±SEM. For each depth layer, we computed a GLM analysis (see Methods). The lines show beta weights averaged across voxels and participants for each condition (separate for each colour) and depth. The dashed black lines designate a mean beta weight of zero for reference; asterisks denote significant above-chance decoding (p_{adj}<.05, FDR-corrected). In both ROIs of experiment 1 (n=16), fMRI BOLD (as determined by beta weights) was significantly above zero in all conditions (all $p_{adj} \leq$ .008), except for in the mock condition, where only green was significant at a cortical depth of 0.9 (padj = .045). In Experiment 2 (n=10), fMRI BOLD activity was significantly above zero only during perception and mental imagery at all cortical depths and for both colours (all p_{adj} < .05, lower plot). None of the other conditions showed significant above-zero fMRI activity (all p_{adj} > .072). Also see **Supplementary Fig. 7, 8** and **9** for individual subject plots. See **Supplementary Note 2** for repeated measures ANOVAs and post-hoc tests to more directly compare our data with the findings of Kok et al.[7]

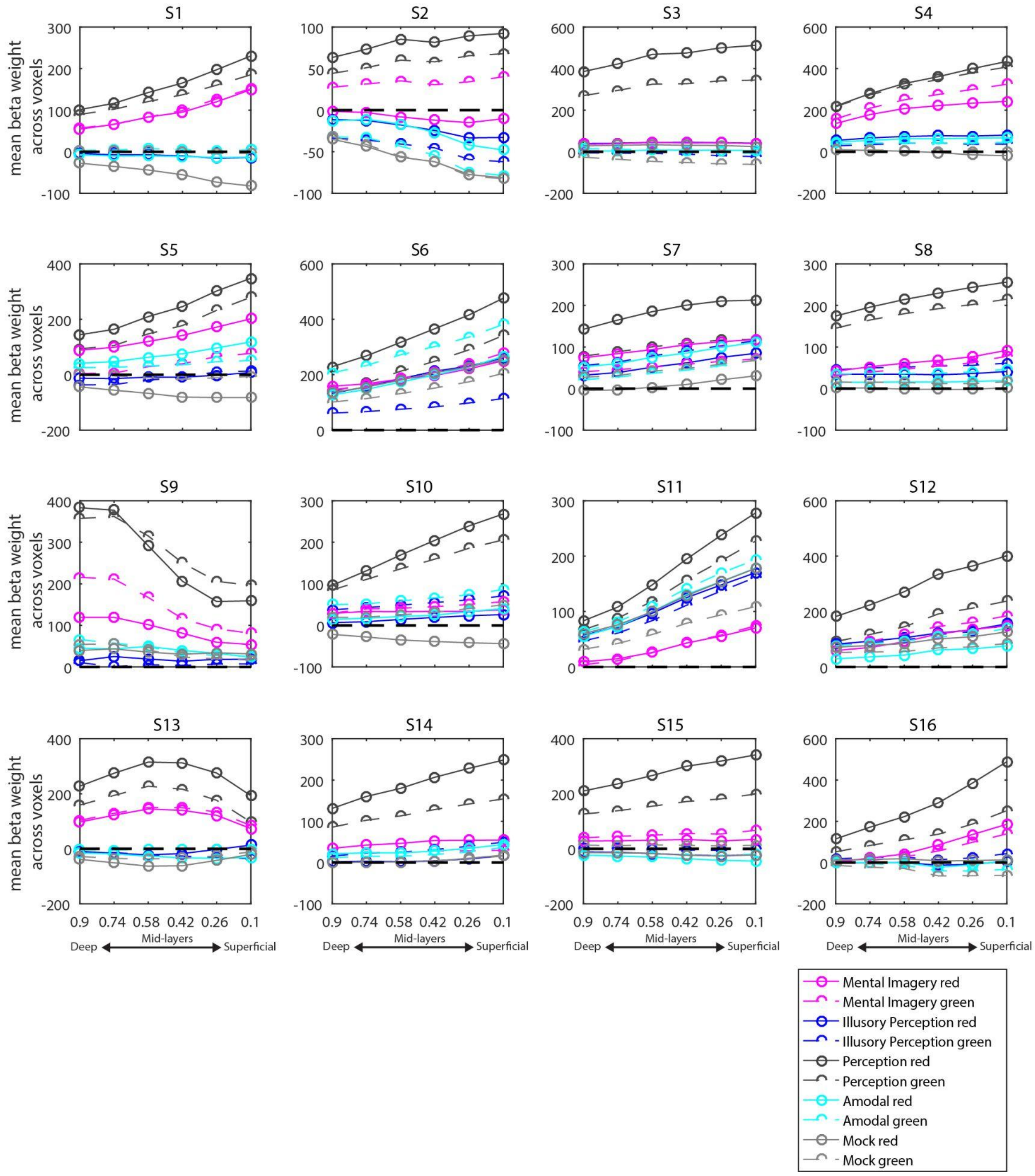

Supplementary Figure 7. Individual fMRI activity plots in the foveal (central) region of interest of Experiment 1 (n=16). In each plot, deeper depth layers (towards the grey matter/white matter boundary) are shown towards the left, and superficial depth layers towards the right (towards the pial surface). Codes above each plot denote the individual subject. Beta weights for each condition were computed in a GLM analysis and then averaged across voxels. The dashed black lines indicate a mean beta weight of zero for reference.

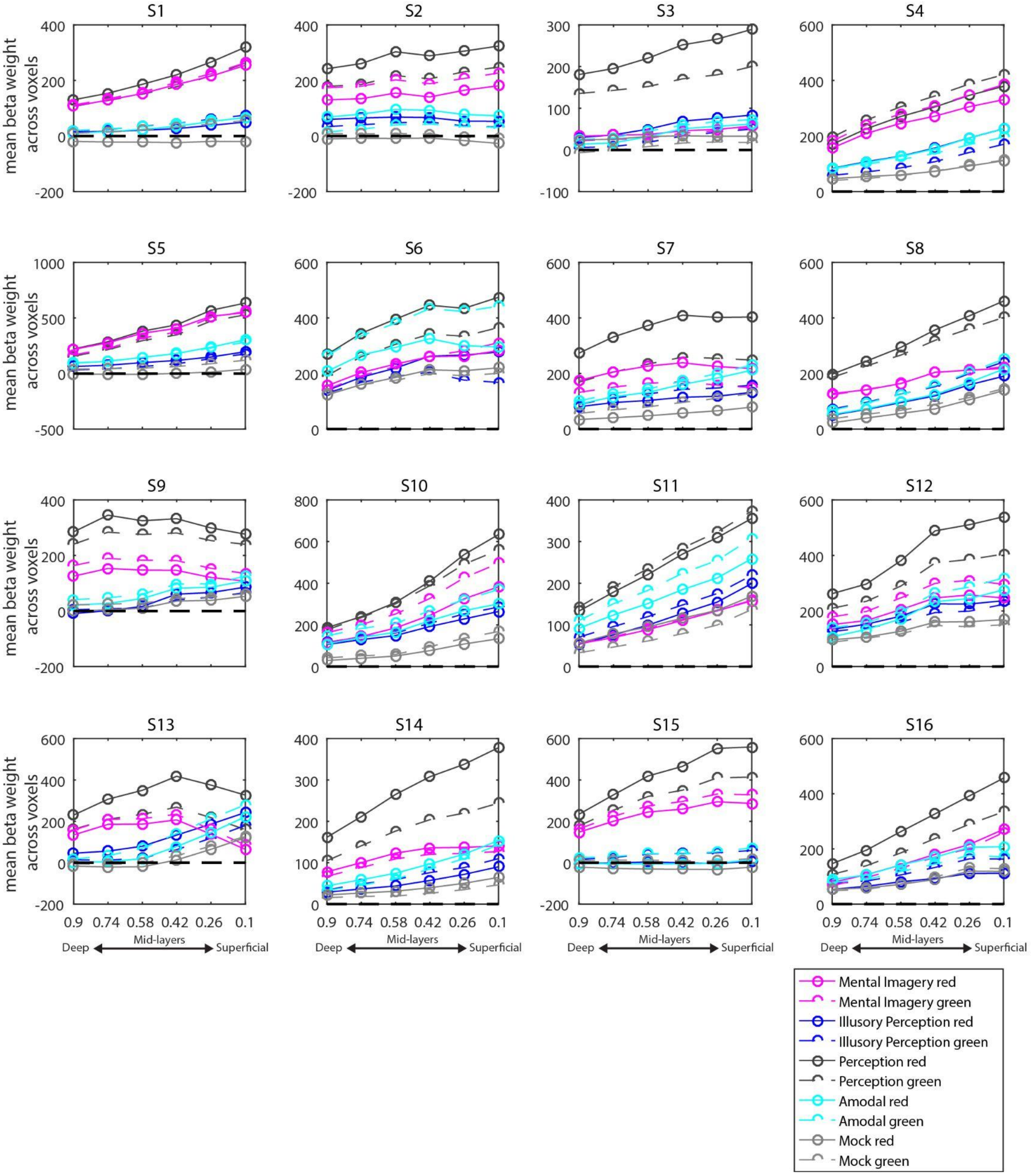

Supplementary Figure 8. Individual fMRI activity plots in the peripheral region of interest (ROI) of Experiment 1 (n=16). In each plot, deep depth layers (towards the grey matter/white matter boundary) are shown on the left, and superficial depth layers on the right (towards the pial surface). Codes above each plot denote the individual subject. Beta weights for each condition were computed in a GLM analysis and then averaged across voxels. The dashed black lines indicate a mean beta weight of zero for reference.

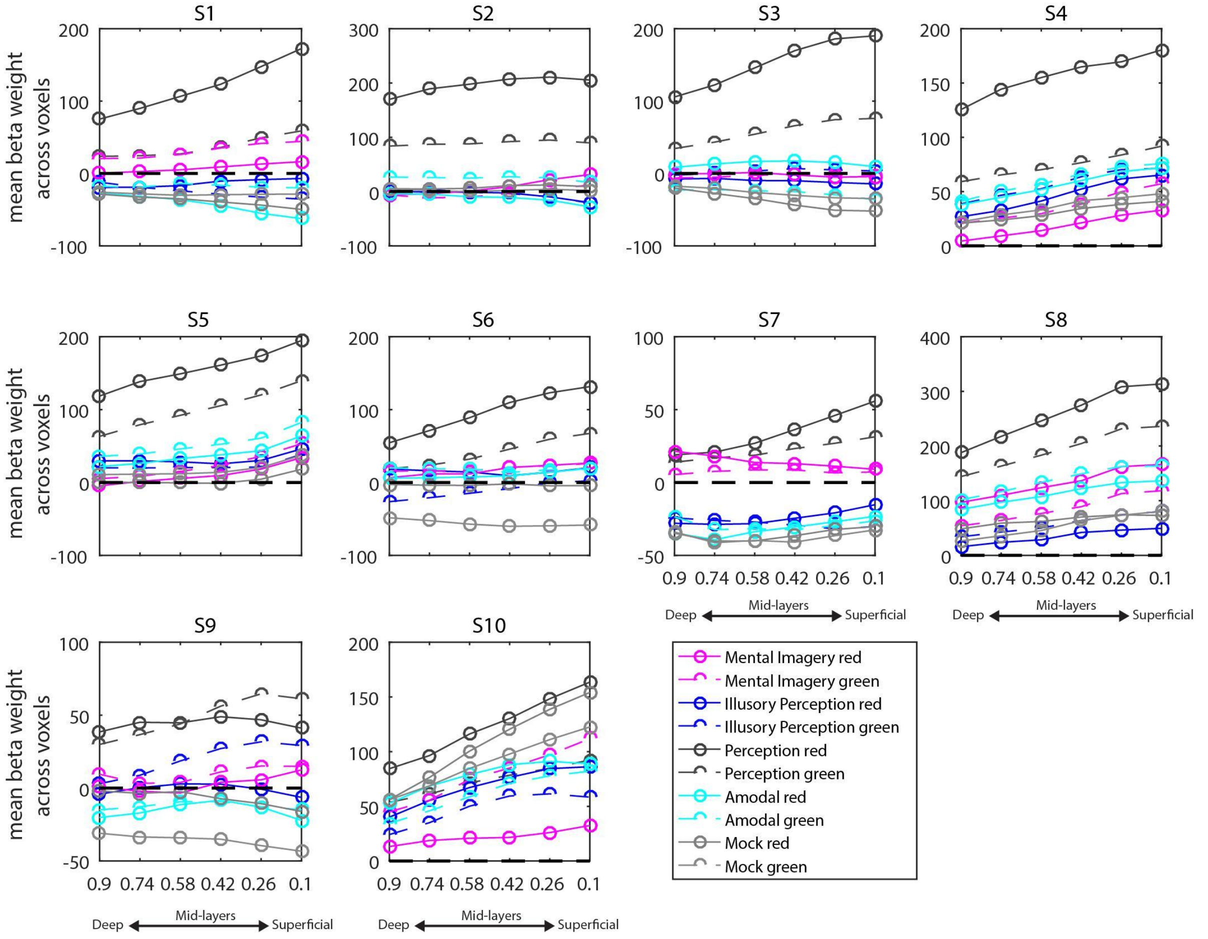

Supplementary Figure 9. Individual fMRI activity plots in the foveal region of interest (ROI) of Experiment 2 (n=10). In each plot, deep depth layers (towards the grey matter/white matter boundary) are shown on the left, and superficial depth layers on the right (towards the pial surface). Codes above each plot denote the individual subject. Beta weights for each condition were computed in a GLM analysis and then averaged across voxels. The dashed black lines indicate a mean beta weight of zero for reference.

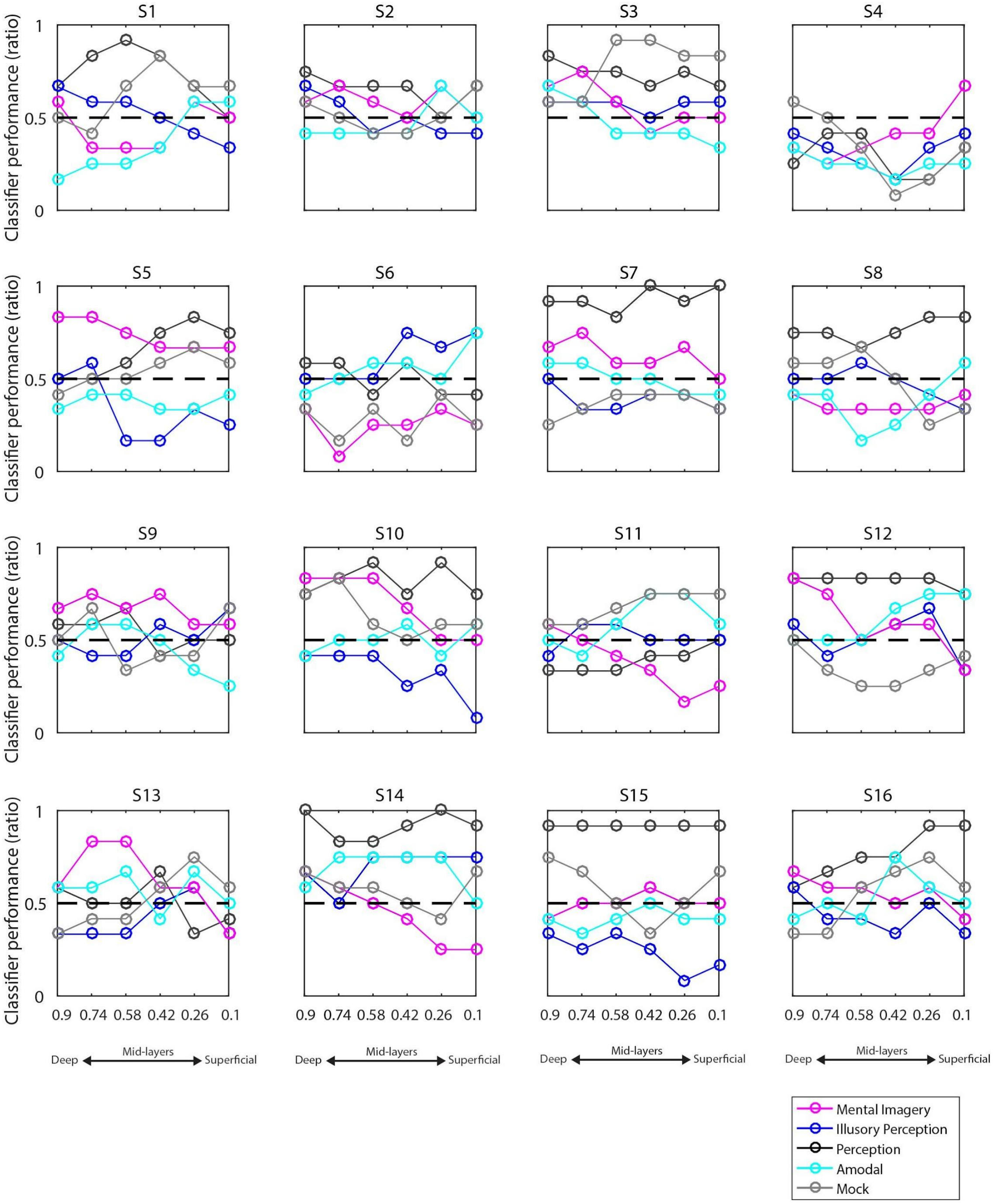

Supplementary Figure 10. Individual decoding plots in the foveal ROI that represents the central portion of the visual field (Experiment 1, n=16). Each plot represents the SVM classification data of one participant. In each plot, deeper depth layers (towards the grey matter/white matter boundary) are shown towards the left, and superficial depth layers towards the right (towards the pial surface). The dashed black lines indicate chance level. Codes above each plot denote the subjects. Note the strong interindividual variability in the laminar decoding profiles, which are common in 7T-fMRI data. It is an open question which factors – apart from unavoidable factors like noise – may contribute to this interindividual variability. We try our best to make sure that the individual data quality is kept to the best standard. Hypothetically, it is plausible to assume that mental imagery ability and the intensity with which individuals perceive visual illusions may contribute to how well its

content may be decodable in the respective layers, as well as how their laminar decoding profile may look like. Whatever the reasons may be, the strong variability highlights the importance of large enough sample sizes and multiple independent tests to ensure that effects at group-level are robust and reliable.

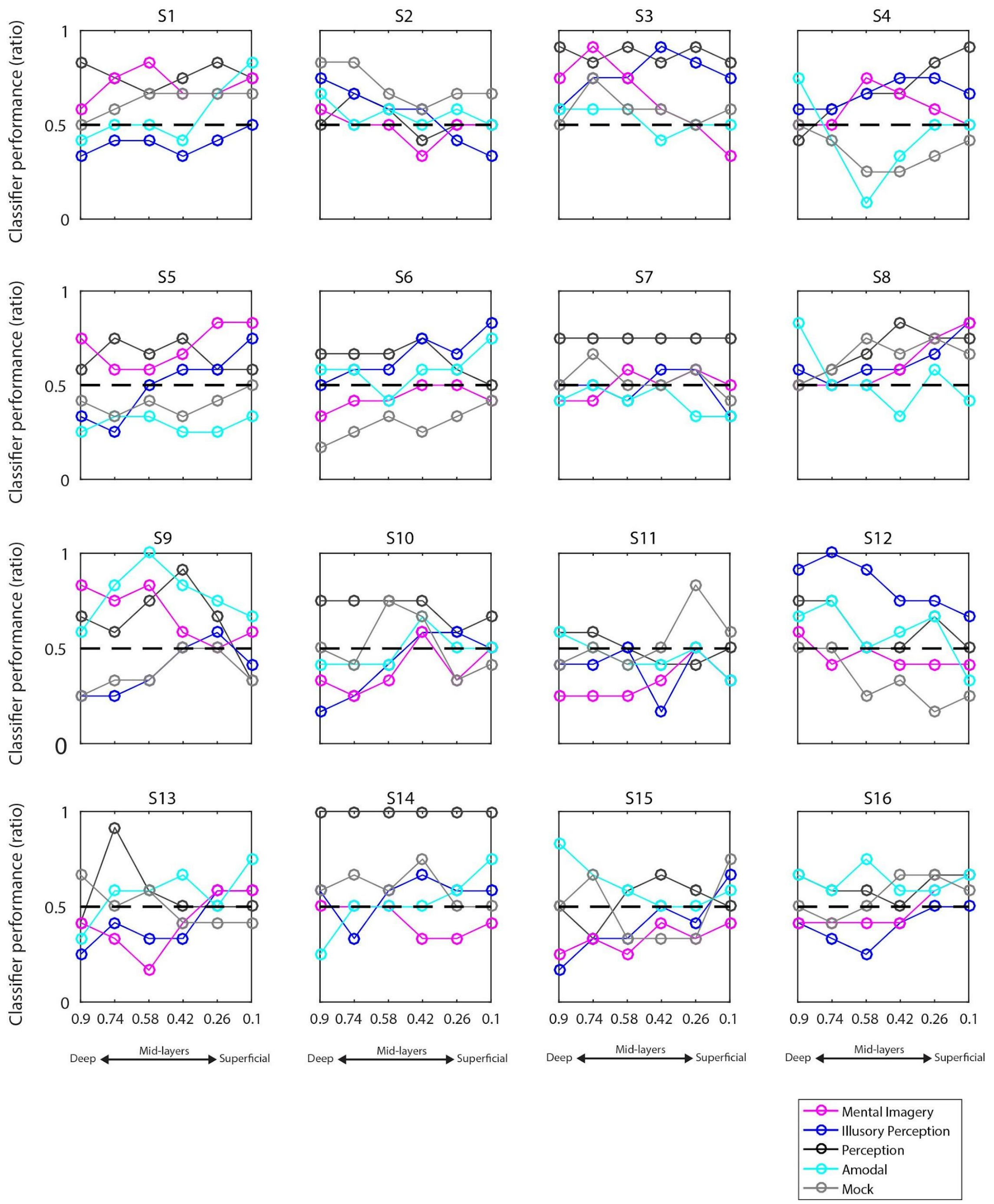

Supplementary Figure 11. Individual decoding plots in the peripheral ROI that represents the peripheral portions of the visual field (Experiment 1, n=16). Each plot represents the SVM classification data of one participant. In each plot, deeper depth layers (towards the grey matter/white matter boundary) are shown towards the left, and superficial depth layers towards the right (towards the pial surface). The dashed black lines indicate chance level. Codes above each plot denote the subjects. Note the strong interindividual variability in the laminar decoding profiles, which are common in 7T-fMRI data. It is an open question which factors – apart from unavoidable factors like noise – may contribute to this interindividual variability. We try our best to make sure that the individual data quality is kept to the best standard. Hypothetically, it is plausible to assume that mental imagery ability and the intensity with which individuals perceive visual illusions may contribute to

how well its content may be decodable in the respective layers, as well as how their laminar decoding profile may look like. Whatever the reasons may be, the strong variability highlights the importance of large enough sample sizes and multiple independent tests to ensure that effects at group-level are robust and reliable.

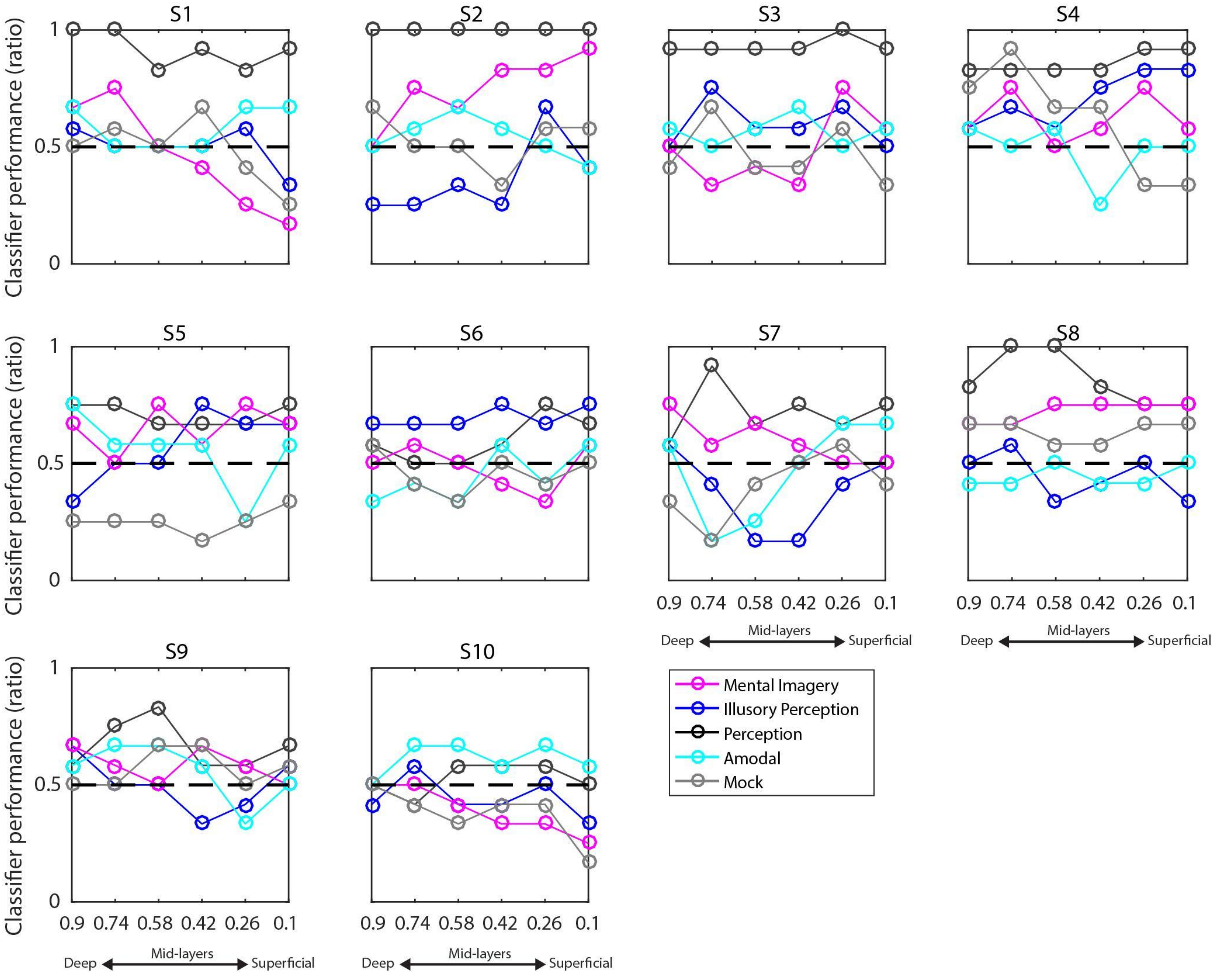

Supplementary Figure 12. Individual decoding plots in the foveal ROI that represents the central portion of the visual field (Experiment 2, n=10). Each plot represents the SVM classification data of one participant. In each plot, deeper depth layers (towards the grey matter/white matter boundary) are shown towards the left, and superficial depth layers towards the right (towards the pial surface). The dashed black lines indicate chance level. Codes above each plot denote the subjects. Note the strong interindividual variability in the laminar decoding profiles, which are common in 7T-fMRI data. It is an open question which factors – apart from unavoidable factors like noise – may contribute to this interindividual variability. We try our best to make sure that the individual data quality is kept to the best standard. Hypothetically, it is plausible to assume that mental imagery ability and the intensity with which individuals perceive visual illusions may contribute to how well its content may be decodable in the respective layers, as well as how their laminar decoding profile may look like. Whatever the reasons may be, the strong variability highlights the importance of large enough sample sizes and multiple independent tests to ensure that effects at group-level are robust and reliable.

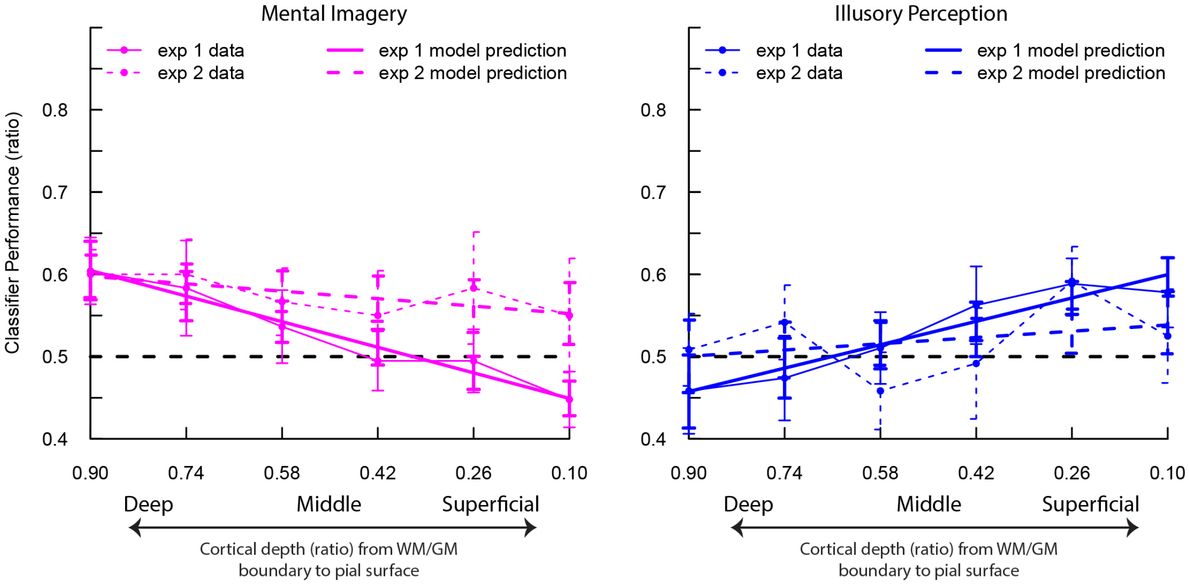

Supplementary Figure 13. Model predictions for individual experiments when a linear mixed model was trained to predict decoding accuracy with depth and stimulus condition for individual experiments 1 and 2 (exp 1 and exp 2; n=16 and n=10), where the regions of interest differed. Model predictions are plotted alongside the experimental data. Mental imagery data and model predictions for both experiments are shown in pink (left), and illusory perception data and model predictions are shown in blue (right).

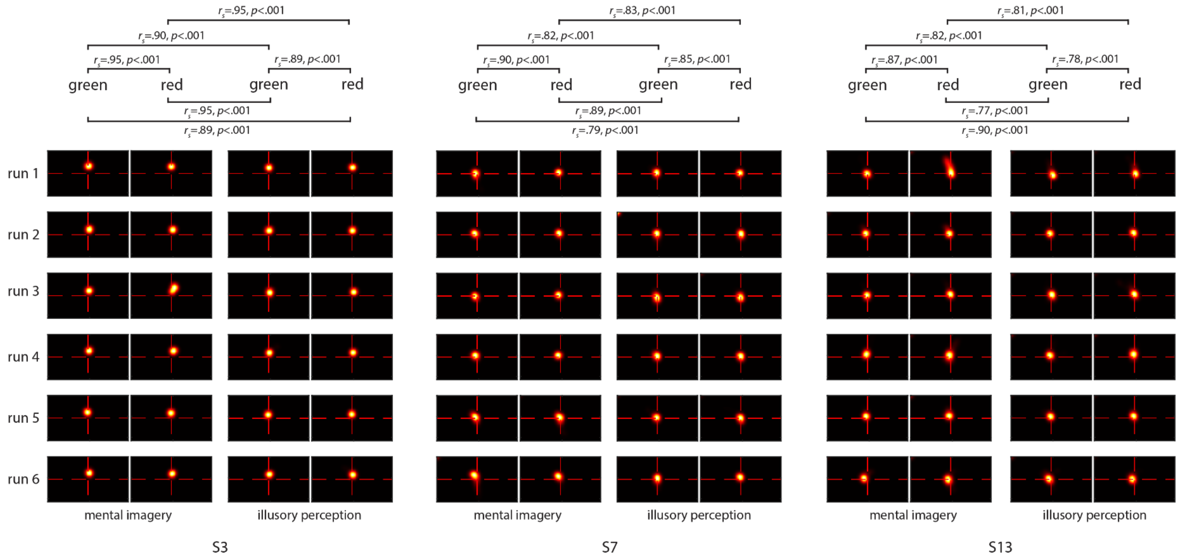

Supplementary Figure 14. Heatmaps of the eye positions for red and green conditions during the illusory perception and imagery trials. Eye tracking data is presented for 3 subjects S3, S7 and S13 of experiment 1 for which we were able to collect eye tracking data for each of the six fMRI runs. To estimate eye position, we used a gaussian kernel with a standard deviation of 1 degree in visual angle around the centre of gaze for each timepoint, and created heatmaps by computing the sum over time during the mental imagery and illusory perception trials (separately for each run). Spearman rank correlations between eye positions during the different conditions and colours are shown above the plots. Overall, eye gaze was highly correlated between conditions and colours in each subject.

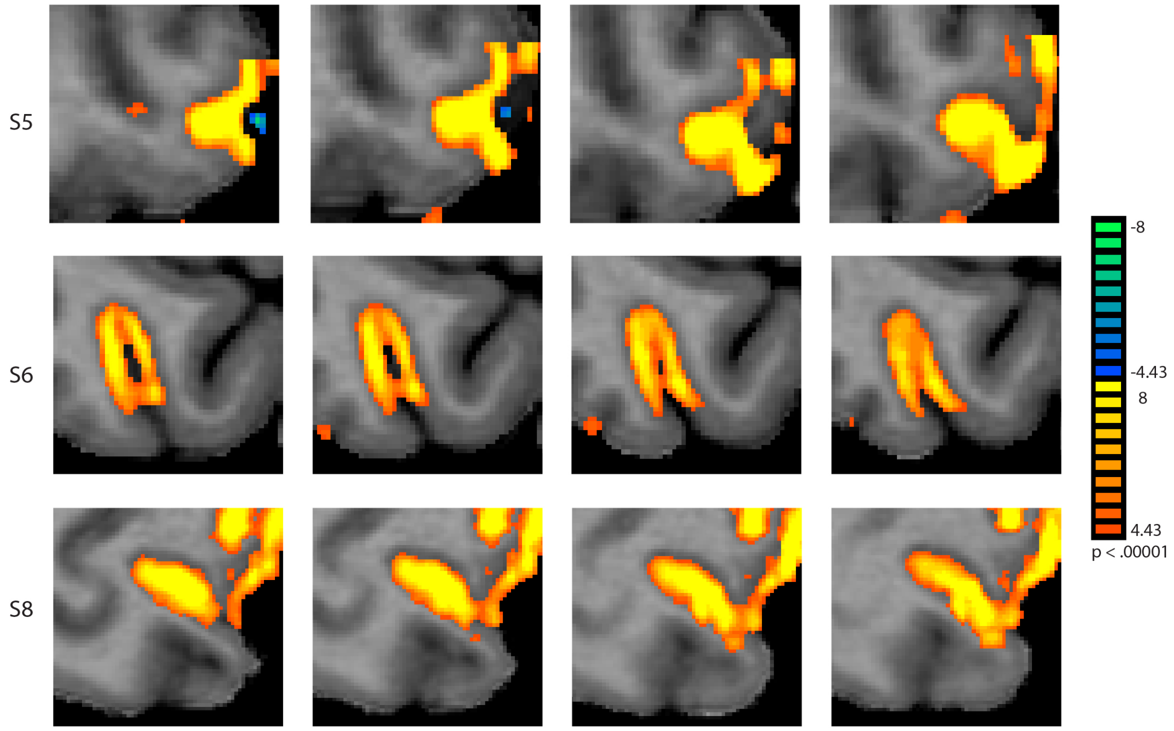

Supplementary Figure 15. Functional MRI images overlayed onto T1-weighted anatomical MRI images. Each row shows consecutive slices of one example subject (all from Experiment 2; right hemisphere, transverse view; slice-direction: upwards). The images show the colour-coded contrast t-maps of the perceptual conditions (red and green) vs. baseline; these elicited the strongest activity among the experimental conditions (see **Supplementary Fig. 6**). To assess the quality of the functional-anatomical alignment, we overlaid the functional onto the anatomical images and inspected their alignment around the ROIs in V1 visually. Note that the functional activity maps were not used to map the ROIs – for this purpose, we used population-receptive field mapping (see Methods and **Supplementary Fig. 2** and **3).**

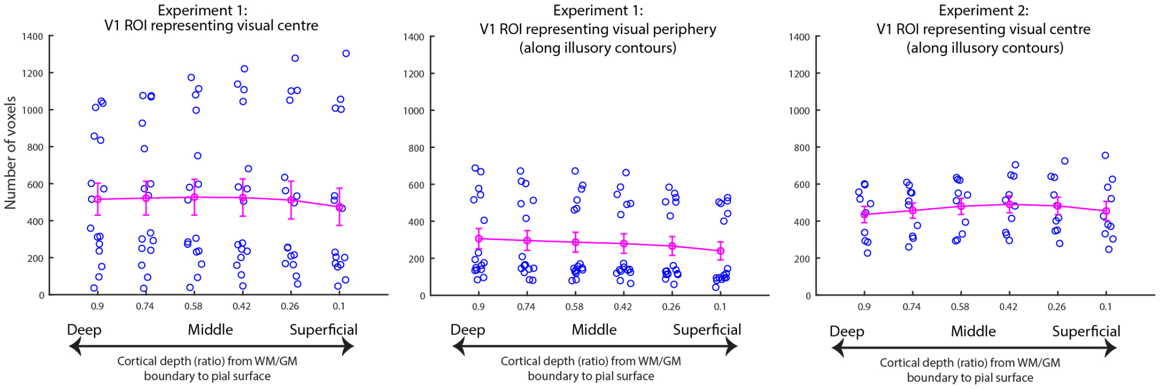

Supplementary Figure 16. Number of voxels across cortical depths in experiment 1 and 2. The blue circles represent the voxel numbers per depth of the individual subjects. The pink lines & circles show the group means across subjects, with error bars denoting ±SEM. Deeper cortical depths are shown towards the left of each plot. There was no significant main effect of depth, indicating that the number of voxels was not significantly different between cortical depth layers ($p > 0.73$). There was also no significant interaction between ROI and depth ($p > .33$).

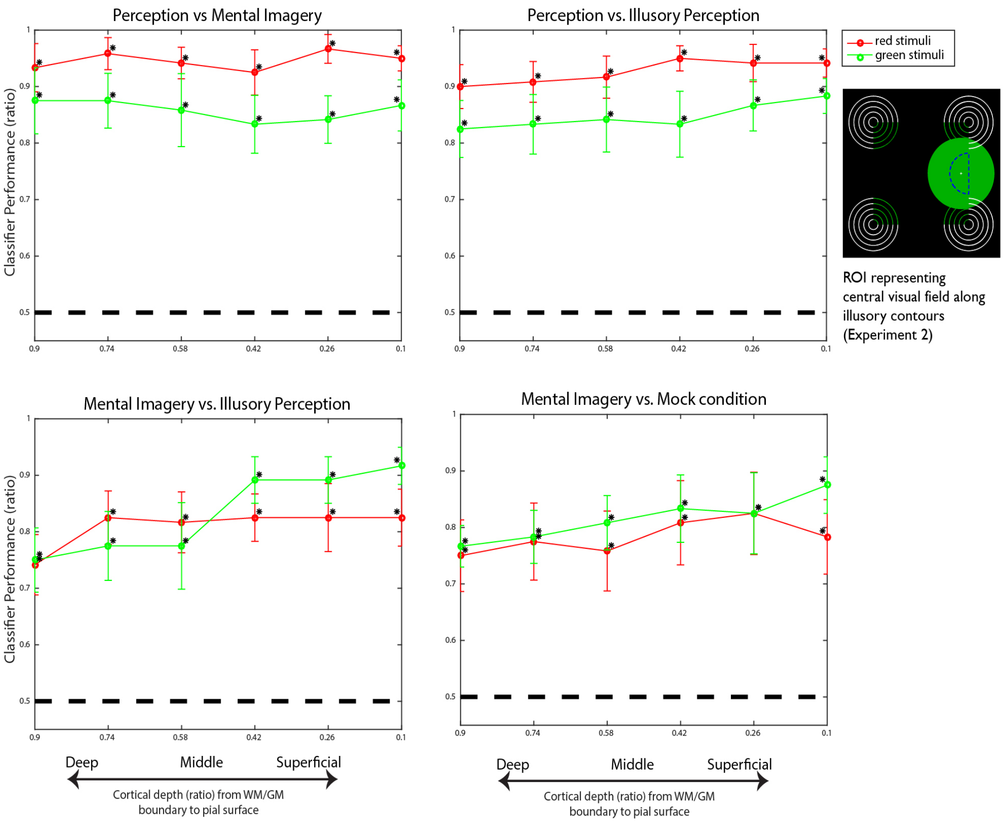

Supplementary Figure 17. Decoding of main conditions against each other. To decode the different experimental conditions against each other, we ran additional SVM classification analyses with the data from experiment 2, where both the illusory contours of the perceptual condition and the imagined and perceptual stimuli were represented within the same V1 region-of-interest. Since all conditions were presented in red and green colour, we could run each analysis twice, one for each colour. Error bars denote ±SEM. Asterisks denote significant above-chance decoding (p_{adj} < .05, FDR-corrected); the dashed black line indicates chance level. When decoding perception vs. mental imagery (first plot), perception vs. illusory perception (second plot), illusory perception vs. mental imagery (third plot), and mental imagery against mock version of the illusory stimulus (fourth plot), decoding is highly significantly above chance for both colours in all layers (all p_{adj} < .001). The classifier results reflect the univariate difference between these conditions (**Supplementary Fig. 6**): Activity levels (as expressed by the across-voxel means of the beta estimates for each condition) differ between conditions across layers (and these differences increase towards superficial layers), making it easy for the SVM classifier to distinguish between different conditions.

Supplementary Notes

Supplementary Note 1

Decoding illusory perception vs. mock condition. As shown in **Supplementary Fig. 4A**, decoding accuracies increased towards the more superficial layers in both colours; in Experiment 1, the red stimuli showed significant decoding in more layers than the green stimuli, but both red and green stimuli showed significant above-threshold decoding in the second and third most superficial depth (at depth 0.42, i.e. 3rd depth - red: $\hat{\mu}$ = 0.67, p_{adj} < .001, 90% CI [0.59, 0.73]; green: $\hat{\mu}$ = 0.63, p_{adj} = .002, 90% CI [0.56, 0.69]; at depth 0.26, i.e. 2nd depth – red: $\hat{\mu}$ = 0.66, p_{adj} < .001, 90% CI [0.59, 0.73], green: $\hat{\mu}$ = 0.59 , p_{adj} = .008, 90% CI [0.54, 0.64]; bootstrapped and FDR-corrected). The pattern of results largely replicated in Experiment 2 (**Supplementary Fig. 4B**), where both colours only show significant decoding in the third cortical depth (red: $\hat{\mu}$ = 0.65, p_{adj} = .02, 90% CI [0.56, 0.74]; green: $\hat{\mu}$ = 0.61, p_{adj} = .02, 90% CI [0.53, 0.68]), and green stimuli in the second cortical depth ($\hat{\mu}$ = 0.59, p_{adj} = .02, 90% CI [0.53, 0.65]); decoding of the red stimuli shows larger variability at the second cortical depth and therefore fails to reach statistical significance here (p_{adj} = 0.14). This pattern of results does not align with Kok's et al.'s results, who found selective deep layer involvement, but is in line with our finding that illusory colour can only be decoded at the second superficial depth, as reported in the main text. It is also supported by findings in non-human primates[22] and mice[21]that edges between figure and ground lead to additional V1 activity caused by synaptic input into layer 2/3.

Supplementary Note 2

Univariate analysis of fMRI BOLD activity levels. In a repeated measures ANOVA, the main effects of cortical depth (F(5,75) = 39.01, p < .001, η^2_G = .084), V1 location (F(1,15) = 52.81, p < .001, η^2_G = .245) and stimulus condition (F(9,135) = 47.73, p < .001, η^2_G = .497) were significant in Experiment 1, indicating that fMRI activity (1) increases towards superficial depths; (2) was higher overall in the peripheral ROI compared to the foveal ROI; and (3) differed between the different stimulus conditions. In addition, all three 2-way interactions and the 3-way interaction between cortical depth, V1 location and stimulus conditions showed significance (all p < .001). The results were similar in Experiment 2, where only one central ROI was used: again, we found significant main effects of cortical depth (F(5,45) = 7.69, p < .001, η^2_G = .015) and stimulus condition (F(9,81) = 17.18, p < .001, η^2_G = .358), and a significant two-way interaction between cortical depth and stimulus condition (F(45,405) = 3.26, p < .001, η^2_G = .01), the latter indicating that the increase in fMRI activity towards more superficial depths was stronger in some conditions compared to others (also see **Supplementary Fig. 6**). The univariate differences between the main stimulation conditions presumably result in high decoding accuracies when decoding the conditions against each other (**Supplementary Fig. 17**).

To more directly compare our data with the findings of Kok et al.[7], we also ran post-hoc tests to compare fMRI BOLD activation levels (as determined by beta weights) during illusory perception against the amodal and mock control versions of the stimulus, separately for the two colours. When comparing illusory perception against the mock condition

(peripheral ROI in experiment 1), we found significant differences across all layers in both the red and green versions of the stimuli (all $p_{adj} < .001$, FDR-corrected). When comparing illusory perception against the amodal condition, we again found significant differences across all layers for the green version of the stimuli (all $p_{adj} < .02$); for the red version, only the more superficial layers were significantly different in activation levels (depths 0.1, 0.26, 0.42; all $p_{adj} < .025$). In the second experiment, there were no significant differences between the illusory perception conditions and either of the two control conditions (all $p_{adj} > .1$). Again, like our main results and the analyses shown in **Supplementary Fig. 4A** and **B,** these results do not replicate previous findings that illusory perception selectively involves deep layers[7].
