# Supplementary material for: Cortical depth profiles in primary visual cortex for illusory and imaginary experiences
Source: Nat Commun. 2024 Feb 2;15:1002. doi: 10.1038/s41467-024-45065-w (PMC10837448; doi:10.1038/s41467-024-45065-w)
Supplement: Supplementary file 3 — Reporting Summary [file 41467_2024_45065_MOESM3_ESM.pdf]

nature portfolio

Corresponding author(s): Johanna Bergmann

Last updated by author(s): October 31, 2023

Reporting Summary

Nature Portfolio wishes to improve the reproducibility of the work that we publish. This form provides structure for consistency and transparency in reporting. For further information on Nature Portfolio policies, see our Editorial Policies and the Editorial Policy Checklist.

Statistics

For all statistical analyses, confirm that the following items are present in the figure legend, table legend, main text, or Methods section.

n/a	Confirmed	
☐	☒	The exact sample size (*n*) for each experimental group/condition, given as a discrete number and unit of measurement
☐	☒	A statement on whether measurements were taken from distinct samples or whether the same sample was measured repeatedly
☐	☒	The statistical test(s) used AND whether they are one- or two-sided<br>*Only common tests should be described solely by name; describe more complex techniques in the Methods section.*
☐	☒	A description of all covariates tested
☐	☒	A description of any assumptions or corrections, such as tests of normality and adjustment for multiple comparisons
☐	☒	A full description of the statistical parameters including central tendency (e.g. means) or other basic estimates (e.g. regression coefficient) AND variation (e.g. standard deviation) or associated estimates of uncertainty (e.g. confidence intervals)
☐	☒	For null hypothesis testing, the test statistic (e.g. *F*, *t*, *r*) with confidence intervals, effect sizes, degrees of freedom and *P* value noted<br>*Give P values as exact values whenever suitable.*
☒	☐	For Bayesian analysis, information on the choice of priors and Markov chain Monte Carlo settings
☐	☒	For hierarchical and complex designs, identification of the appropriate level for tests and full reporting of outcomes
☐	☒	Estimates of effect sizes (e.g. Cohen's *d*, Pearson's *r*), indicating how they were calculated

Our web collection on statistics for biologists contains articles on many of the points above.

Software and code

Policy information about availability of computer code

Data collection	We used MatlabR2016a R2016 and Psychtoolbox v3.0.13 for experimental stimulus presentation.
Data analysis	MATLAB R2015b & R2016 in connection with LIBSVM, v2.86, BVQX v0.8b /NeuroElf v0.9c toolboxes, as well as R (rstatix, nlme, bestNormalize, report packages). The code of this study is available at https://gitlab.com/joebee/7t-imagery-illusory-in-v1-layers

For manuscripts utilizing custom algorithms or software that are central to the research but not yet described in published literature, software must be made available to editors and reviewers. We strongly encourage code deposition in a community repository (e.g. GitHub). See the Nature Portfolio guidelines for submitting code & software for further information.

Data

Policy information about availability of data

All manuscripts must include a data availability statement. This statement should provide the following information, where applicable:

- Accession codes, unique identifiers, or web links for publicly available datasets
- A description of any restrictions on data availability
- For clinical datasets or third party data, please ensure that the statement adheres to our policy

The data that support the findings of this study are available on the Ebrains Knowledge Graph, https://kg.ebrains.eu/search/instances/Dataset/de7a6c44-8167-44a8-9cf4-435a3dab61ed

Research involving human participants, their data, or biological material

Policy information about studies with human participants or human data. See also policy information about sex, gender (identity/presentation), and sexual orientation and race, ethnicity and racism.

Reporting on sex and gender	Gender was determined based on self-report. All subjects gave informed consent regarding the pseudonymized use of their data. No gender-based analyses were performed as there were no gender-specific hypotheses regarding the processing of information in the different layers of primary visual cortex.
Reporting on race, ethnicity, or other socially relevant groupings	Apart from gender and age we did not collect any other demographical data (like race, ethnicity or other socially relevant groupings).
Population characteristics	fMRI data were collected from 18 healthy participants in Experiment 1 (age range 19-36 years at the time of the experiment, M=23.3, SD=4.96; 14 females and 4 males), and 12 healthy participants in Experiment 2 (age range 19-31 years at the time of the experiment, M=23.2, SD=4.02; 8 females and 4 males). All had normal or corrected-to-normal vision. The participants were recruited from a larger participant sample on the basis of how they scored on an imagery strength measure quantified in a behavioural pre-test. This pre-test sample consisted of 55 individuals in Experiment 1 (age range 18-36 years; 20 males, 35 females) and 52 individuals in Experiment 2 (age range 18-33 years, M=23.1, SD=3.76, 18 males, 34 females). Four of the participants of Experiment 2 had already participated in Experiment 1, and therefore did not do the pre-test again. Of the pre-sample of Experiment 1, 21 participants had an imagery strength score that met or surpassed an a priori defined threshold, and were invited to take part in the main fMRI experiment. 18 of these completed the fMRI experiment. For Experiment 2, 11 participants surpassed the a priori defined threshold (plus the 4 participants who had already taken part in Experiment 1, i.e. 15 in total). 12 of them completed the fMRI experiment.
Recruitment	Participants were recruited from the participant pool of the Institute of Neuroscience and Psychology, University of Glasgow via adverts on the Institute's online study recruitment system. In the advert, potential participants were informed that the study was about visual imagery and that we were particularly looking for individuals with strong imagery ability.
Ethics oversight	The study was approved by the ethics committee of the College of Science and Engineering and the College of Medical, Veterinary and Life Sciences of the University of Glasgow.

Note that full information on the approval of the study protocol must also be provided in the manuscript.

Field-specific reporting

Please select the one below that is the best fit for your research. If you are not sure, read the appropriate sections before making your selection.

☒ Life sciences ☐ Behavioural & social sciences ☐ Ecological, evolutionary & environmental sciences

For a reference copy of the document with all sections, see nature.com/documents/nr-reporting-summary-flat.pdf

Life sciences study design

All studies must disclose on these points even when the disclosure is negative.

Sample size	No formal power analysis was conducted to pre-determine sample size as the study is exploratory, but the number of participants exceeds that of most other V1 laminar fMRI studies currently published. We carried out a two experiments, the second one of which represented a conceptual replication of the first experiment. fMRI data were collected from 18 healthy participants in Experiment 1 (age range 19-36 years at the time of the experiment, M=23.3, SD=4.96; 14 females and 4 males), and 12 healthy participants in Experiment 2 (age range 19-31 years at the time of the experiment, M=23.2, SD=4.02; 8 females and 4 males). All had normal or corrected-to-normal vision. The participants were recruited from a larger participant sample on the basis of how they scored on an imagery strength measure quantified in a behavioural pre-test. This pre-test sample consisted of 55 individuals in Experiment 1 (age range 18-36 years; 20 males, 35 females) and 52 individuals in Experiment 2 (age range 18-33 years, M=23.1, SD=3.76, 18 males, 34 females). Four of the participants of Experiment 2 had already participated in Experiment 1, and therefore did not do the pre-test again. Of the pre-sample of Experiment 1, 21 participants had an imagery strength score that met or surpassed an a priori defined threshold, and were invited to take part in the main fMRI experiment. 18 of these completed the fMRI experiment. For Experiment 2, 11 participants surpassed the a priori defined threshold (plus the 4 participants who had already taken part in Experiment 1, i.e. 15 in total). 12 of them completed the fMRI experiment.
Data exclusions	nter-run alignment had to be at least r>.9 on average (a priori set threshold). The data of 2 of the 18 participants showed very bad inter-run alignment (r=.13 and r=.38, respectively). After several failed attempts to improve the alignment, the two data sets were removed from further analysis. Similarly, in Experiment 2, 2 of the 12 participants showed below-threshold inter-run alignment, and one of these two also showed no discernible retinotopic maps in either of the two hemispheres. Although the initial alignment was not as low as that of the two participants in Experiment 1 (r = .72 and r = .85), multiple attempts to improve alignment enough to pass the a priori-defined threshold of r>.9 failed, and therefore the two data sets were removed from further analysis.
Replication	Experiment 2 represents one conceptual replication of Experiment 1. Furthermore, each of the experiments contains several independent tests that replicate the main findings.
Randomization	Within-subject design, so no allocation to different experimental groups. Sequence order of stimulus presentation was randomized.
Blinding	Not applicable, as a within-subject design was used and participants were not allocated to different experimental groups.

Reporting for specific materials, systems and methods

We require information from authors about some types of materials, experimental systems and methods used in many studies. Here, indicate whether each material, system or method listed is relevant to your study. If you are not sure if a list item applies to your research, read the appropriate section before selecting a response.

Materials & experimental systems

n/a	Involved in the study
☒	☐ Antibodies
☒	☐ Eukaryotic cell lines
☒	☐ Palaeontology and archaeology
☒	☐ Animals and other organisms
☒	☐ Clinical data
☒	☐ Dual use research of concern
☒	☐ Plants

Methods

n/a	Involved in the study
☒	☐ ChIP-seq
☒	☐ Flow cytometry
☐	☒ MRI-based neuroimaging

Plants

Seed stocks

Report on the source of all seed stocks or other plant material used. If applicable, state the seed stock centre and catalogue number. If plant specimens were collected from the field, describe the collection location, date and sampling procedures.

Novel plant genotypes

Describe the methods by which all novel plant genotypes were produced. This includes those generated by transgenic approaches, gene editing, chemical/radiation-based mutagenesis and hybridization. For transgenic lines, describe the transformation method, the number of independent lines analyzed and the generation upon which experiments were performed. For gene-edited lines, describe the editor used, the endogenous sequence targeted for editing, the targeting guide RNA sequence (if applicable) and how the editor was applied.

Authentication

Describe any authentication procedures for each seed stock used or novel genotype generated. Describe any experiments used to assess the effect of a mutation and, where applicable, how potential secondary effects (e.g. second site T-DNA insertions, mosiacism, off-target gene editing) were examined.

Magnetic resonance imaging

Experimental design

Design type

Block design (task-based)

Design specifications

1 fMRI scanning session. 6 experimental runs, 30 trials each (plus 4 trials for central target area + surround mapping); hence, 30 trials for 5 conditions = 6 trials per condition (3 x red & 3 x green stimuli) per run. Additionally: retinotopic mapping runs (polar angle and eccentricity mapping, one run each).

Behavioral performance measures

N/A

Acquisition

Imaging type(s)

functional

Field strength

7 Tesla

Sequence & imaging parameters

gradient-echo EPI using the CMRR MB sequence with an MB factor of 1 (voxel resolution: 0.8 x 0.8 x 0.8mm3 isotropic resolution, distance factor: 0%, 27 slices, FoV=148mm, TR= 2000 ms, TE=26.4 ms, flip angle: 70°, slice timing: interleaved, bandwidth = 1034Hz/px, phase encoding direction: head to foot)

Area of acquisition

occipital cortex, EPI slab aligned to capture calcarine sulcus where V1 is located

Diffusion MRI ☐ Used ☒ Not used

Preprocessing

Preprocessing software

Functional data were preprocessed using BrainVoyager 20.6

Normalization

rigid-body transformation to ACPC space

Normalization template

ACPC space

Noise and artifact removal	motion correction, high-pass filtering, slice-time correction
Volume censoring	N/A

Statistical modeling & inference

Model type and settings	multivariate pattern analysis (SVM classification)
Effect(s) tested	To test the mean SVM classification accuracy across subjects for each stimulus condition at each cortical depth against chance level (50%), we used bootstrapping of the mean with 10,000 samples. The statistical significance level of α=0.05 (one-sided) was corrected for multiple comparisons using false discovery rate (FDR)

Specify type of analysis: ☐ Whole brain ☒ ROI-based ☐ Both

Anatomical location(s): ROIs in V1 representing certain portions in visual field (determined via population-receptive field mapping)

Statistic type for inference (See Eklund et al. 2016)	bootstrapping of the mean with 10,000 samples, one-sided
Correction	FDR

Models & analysis

n/a	Involved in the study
☒	☐ Functional and/or effective connectivity
☒	☐ Graph analysis
☐	☒ Multivariate modeling or predictive analysis

Multivariate modeling and predictive analysis

For the multivariate pattern analysis, a linear SVM classification with default parameters was computed. Voxels whose raw mean BOLD signal intensities were below 100 were removed prior to the analysis. Beta weights for every trial were then estimated in a GLM analysis. Before entering the data into the SVM classifier, the beta weights were normalized by rescaling the values between -1 and 1. Classification models for the different depths were trained using the C-SVM method (cost parameter = 1) with a linear kernel, implemented in the LIBSVM toolbox, v2.86. Tolerance for termination was 0.001 (default setting) and cost parameters were equal across classes (i.e. no applied weighting scheme for the different conditions). Cross-validation was performed in a leave-one-run-out manner. The reported SVM accuracies were averaged across cross-validation folds.

In a second-level analysis, we fitted a linear mixed model (estimated using REML and nlminb optimizer) to predict decoding accuracy with depth, experiment and stimulus condition. This was done to compare the two critical conditions - mental imagery and illusory perception - more directly at the different cortical depths. Computing a linear mixed model has the advantage of avoiding the multiplication of tests and therefore multiple comparisons by using parametrization across layers and between experiments. This enables us to harvest the power of accumulating evidence. The parametrization across cortical depth was possible because the number of cortical depths we define is arbitrary, these depths are partially overlapping and they only have a statistical correspondence with histology. The second-level analysis approach also allowed us to examine any statistical differences between the two experiments. To compute the model, we first pooled the data of the critical conditions from the two experiments - i.e. mental imagery decoding in the central ROI and illusory perception decoding in the peripheral ROI from experiment 1, and mental imagery and illusory perception decoding in the central ROI from experiment 2. We included as predictors an intercept, depth, experiment, stimulus condition, the interaction between experiment and depth, between stimulus condition and depth, between experiment, stimulus condition and depth. As random effects across participants, we included intercept, depth and the interaction between stimulus condition and depth.
